# An interactive national digital surveillance system to fight against COVID-19 in Bangladesh

**DOI:** 10.3389/fdgth.2023.1059446

**Published:** 2023-05-11

**Authors:** Farhana Sarker, Moinul H. Chowdhury, Ishrak Jahan Ratul, Shariful Islam, Khondaker A. Mamun

**Affiliations:** ^1^ CMED Health Ltd., Dhaka, Bangladesh; ^2^ Department of CSE, University of Liberal Arts, Dhaka, Bangladesh; ^3^ Advanced Intelligent Multidisciplinary Systems Lab (AIMS Lab), United International University, Dhaka, Bangladesh; ^4^ School of Exercise & Nutrition Sciences, Faculty of Health, Deakin University, Burwood, VIC, Australia; ^5^ Department of CSE, United International University, Dhaka, Bangladesh

**Keywords:** COVID-19, artificial intelligence, screening, digital health, surveillance system

## Abstract

**Background:**

COVID-19 has affected many people globally, including in Bangladesh. Due to a lack of preparedness and resources, Bangladesh has experienced a catastrophic health crisis, and the devastation caused by this deadly virus has not yet been halted. Hence, precise and rapid diagnostics and infection tracing are essential for managing the condition and limiting its spread. The conventional screening procedure, such as reverse transcription polymerase chain reaction (RT-PCR), is not available in most rural areas and is time-consuming. Therefore, a data-driven intelligent surveillance system can be advantageous for rapid COVID-19 screening and risk estimation.

**Objectives:**

This study describes the design, development, implementation, and characteristics of a nationwide web-based surveillance system for educating, screening, and tracking COVID-19 at the community level in Bangladesh.

**Methods:**

The system consists of a mobile phone application and a cloud server. The data is collected by community health professionals *via* home visits or telephone calls and analyzed using rule-based artificial intelligence (AI). Depending on the results of the screening procedure, a further decision is made regarding the patient. This digital surveillance system in Bangladesh provides a platform to support government and non-government organizations, including health workers and healthcare facilities, in identifying patients at risk of COVID-19. It refers people to the nearest government healthcare facility, collecting and testing samples, tracking and tracing positive cases, following up with patients, and documenting patient outcomes.

**Results:**

This study began in April 2020, and the results are provided in this paper till December 2022. The system has successfully completed 1,980,323 screenings. Our rule-based AI model categorized them into five separate risk groups based on the acquired patient information. According to the data, around 51% of the overall screened populations are safe, 35% are low risk, 9% are high risk, 4% are mid risk, and the remaining 1% is very high risk. The dashboard integrates all collected data from around the nation onto a single platform.

**Conclusion:**

This screening can help the symptomatic patient take immediate action, such as isolation or hospitalization, depending on the severity. This surveillance system can also be utilized for risk mapping, planning, and allocating health resources to more vulnerable areas to reduce the virus's severity.

## 1. Introduction

The novel coronavirus disease (COVID-19) was detected in China in December 2019 (1), and as of August 13, 2022, it has caused more than 594.3 million cases and 6.5 million deaths in 213 countries and territories worldwide (2). To prevent the spread of COVID-19, several countries have implemented border restrictions, social distancing, and promoting hand cleanliness, among other measures (3, 4). However, COVID-19 is spreading at an alarming rate throughout several countries (5). The disease's asymptomatic nature further facilitates its acceleration; for example, an infected person may not show any symptoms of COVID-19 for up to nearly 15 days while still infecting others in their contact (6–8). This renders the tracking and tracing of infected persons a problematic task. Human-to-human transmission of the virus occurs in several ways (e.g., droplets and aerosols) (9) and is prevalent in populations with high proximity and a high contact rate. Moreover, South Asian countries are prone to the virus's transmission due to many factors, including high population density, poor hygiene habits, low health literacy, and fragmented health systems (10).

Bangladesh is one of the countries with a very high population density in the world, with a fragile healthcare system, limited resources, and low health awareness among its population. The lack of hand hygiene habits, healthcare preparation, and social isolation has contributed to Bangladesh's rapid spread of COVID-19 (11). There were 2 million confirmed cases and 29,312 deaths in the country as of August 13, 2022 (12), and the number of cases is increasing daily. However, due to a lack of testing facilities, these figures may not accurately depict the disease's spread in Bangladesh (11). Furthermore, testing centers are primarily centered in the capital city, Dhaka, and other major cities. Hence, remote communities with poor healthcare infrastructure may be overlooked in identifying, tracing, and limiting potential COVID-19 infections. To prevent the transmission of the virus, it is necessary to identify and test high-risk patients, trace the contacts of positive cases, isolate and monitor COVID-19 patients, and do contact tracing.

Participatory surveillance and collaborative information sharing technologies have been effectively employed to supplement traditional health surveillance routines in public health surveillance and emergencies (13–20). These approaches provide a rapid pathway to collecting data and monitoring individuals with infectious disease symptoms (15). With the emergence of the COVID-19 pandemic and the lack of pharmacological interventions, many countries have deployed such technologies, including digital surveillance, tracking, and monitoring systems, to combat COVID-19 (21, 22). These technologies included smartphones for contact tracing and isolation, innovative data collection tools, intelligent data analytics, predicting high-risk zones, and data-driven resource mapping to monitor and track COVID-19 cases (23). The introduction of mobile health (mHealth) technology aided in the control of COVID-19. During the COVID period, these mHealth platforms were widely used and performed admirably in early detection, fast screening, patient monitoring, awareness, and treatment to reduce the spread of the virus (24, 25). Additionally, IoT-based platforms were useful for tracking COVID-19 risks (26). Cloud computing systems have been used to monitor, acquire, and store information and to make healthcare systems more efficient and easier to operate (27, 28). Moreover, an AI-driven surveillance platform could aid in case identification and tracking, resource mobilization, and connecting people with care providers. Implementing AI techniques like machine learning and deep learning can be crucial for rapid diagnosis and screening (29). This AI techniques may be beneficial for contact tracking and continuous monitoring of COVID-19 affected individuals (30–34). However, most advanced technology-enabled surveillance systems have been developed and deployed in high-income countries, leaving Bangladesh behind. In response to this ongoing public health emergency, we collaborated with the Community Based Health Care, Directorate General of Health Services, and Ministry of Health and Family Welfare of Bangladesh to develop a countrywide rule-based AI surveillance system. Our system aims to provide community education and awareness about COVID-19, screening prospective COVID-19 patients based on their symptoms, triage, referral, follow-up, monitoring, and case management by mobilizing community health workers in urban and rural Bangladesh. This paper discusses this surveillance system's design, development, and operational characteristics.

## 2. Materials and method

### 2.1. System design

**Figure 1:**
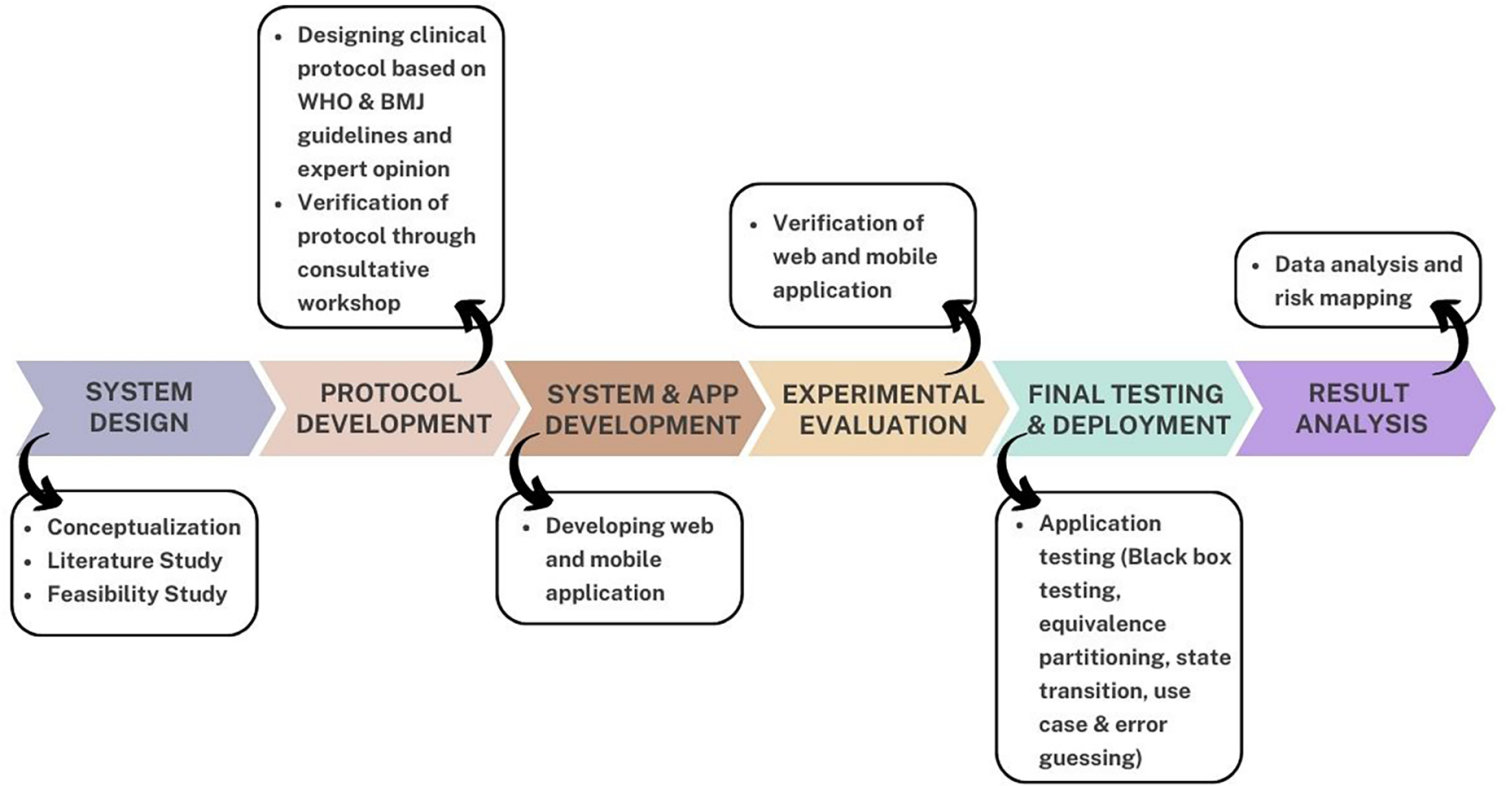
Overall workflow diagram.

The rapid impact of COVID-19 and the use of digital surveillance systems around the world motivated the conceptualization of this study. The COVID-19 digital surveillance system was developed using the COVID-19 World Health Organization (WHO) guidelines (35) and British Medical Journal (BMJ) best practices guidelines (36). Furthermore, thorough analysis and expert opinion are used when designing the protocol. Following the protocol's creation, the system was developed as a web and mobile application. This system was designed to screen the wider populace and obtain statistics for the government and policymakers. The application was released for public usage following rigorous testing and evaluation. The screening results are displayed in the application and web-based dashboard. The dashboard merges all data collected across the country into a single platform. It analyzes data using rule-based AI to deliver insights to crucial government and private sector entities so that they can take the required steps to prevent COVID-19 from spreading throughout Bangladesh. Stakeholders can monitor the screening results in real-time *via* the dashboard, including the total number of persons screened in five risk categories: very high risk, high risk, medium risk, low risk, and no risk. This system's data will be very useful for further pandemic management and assuring the primary healthcare facility in Bangladesh. Figure 1 depicts the overall process for the design, development and application of the surveillance system.

#### 2.1.1. Data source

**Figure 2:**
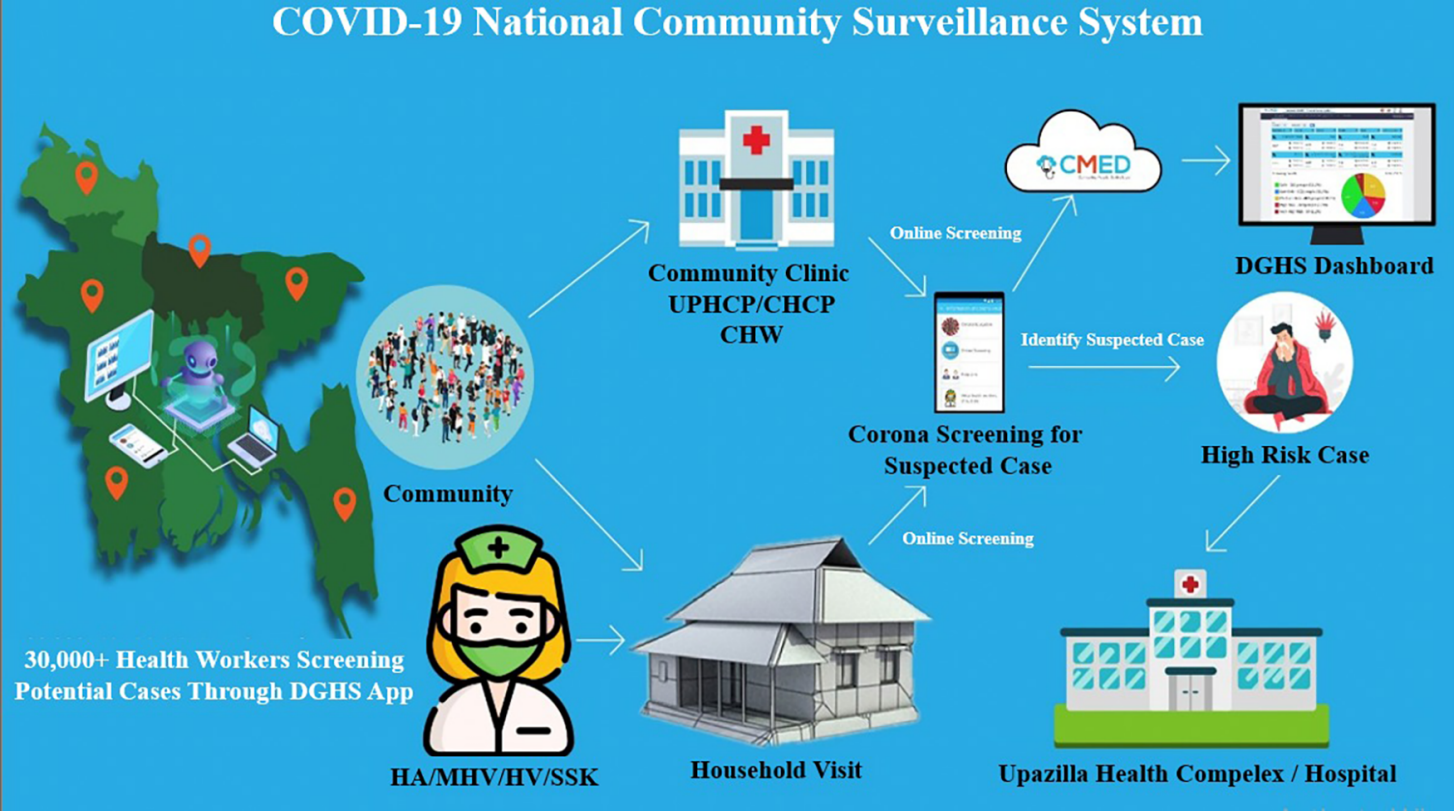
Schematic diagram of the surveillance system.

**Figure 3:**
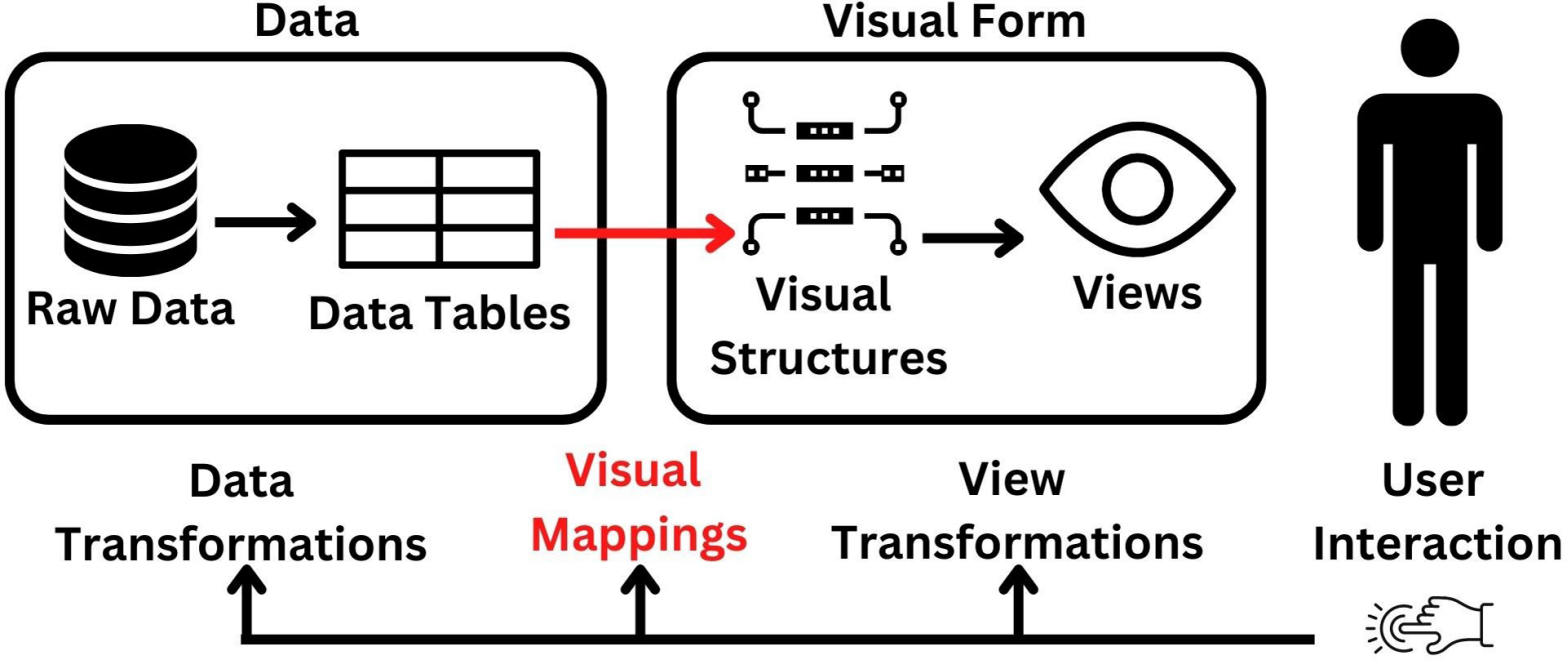
Visual reference model of dataflow.

The current healthcare systems connect community healthcare professionals who conduct household screenings with community clinics and the cloud *via* a mobile phone application. The screening service is accessible to all residents of Bangladesh who are interested in using it, whether they live in urban or rural locations. If a person requests the service, healthcare professionals or community workers can deliver it to the community clinic and their doorstep. Additionally, individuals might receive the service by calling the health professional and providing basic information over the phone. The entire screening data is kept in a safe cloud platform. Depending on the severity of their conditions, people are referred to the next level of care, such as community clinics, Upazila (a sub-unit of a district in Bangladesh) health complexes, and district hospitals. Additionally, data are kept in the cloud and distributed to the appropriate parties for policy making. The users of the dashboards are provided with a username and password so they can browse the information and download various reports. Figure 2 represents the surveillance system's schematic diagram, and Figure 3 illustrates the data flow.

#### 2.1.2. Screening protocol

This study's target sample consisted of individuals with COVID-19 symptoms who were willing to undergo screening and owned or had access to a smartphone. Additionally, those with access to health professionals might utilize the system and undergo screening. Consequently, persons of all ages and genders could participate in this study. Moreover, the system is accessible throughout Bangladesh. This facility was accessible to people from every region, even remote areas.

**Figure 4:**
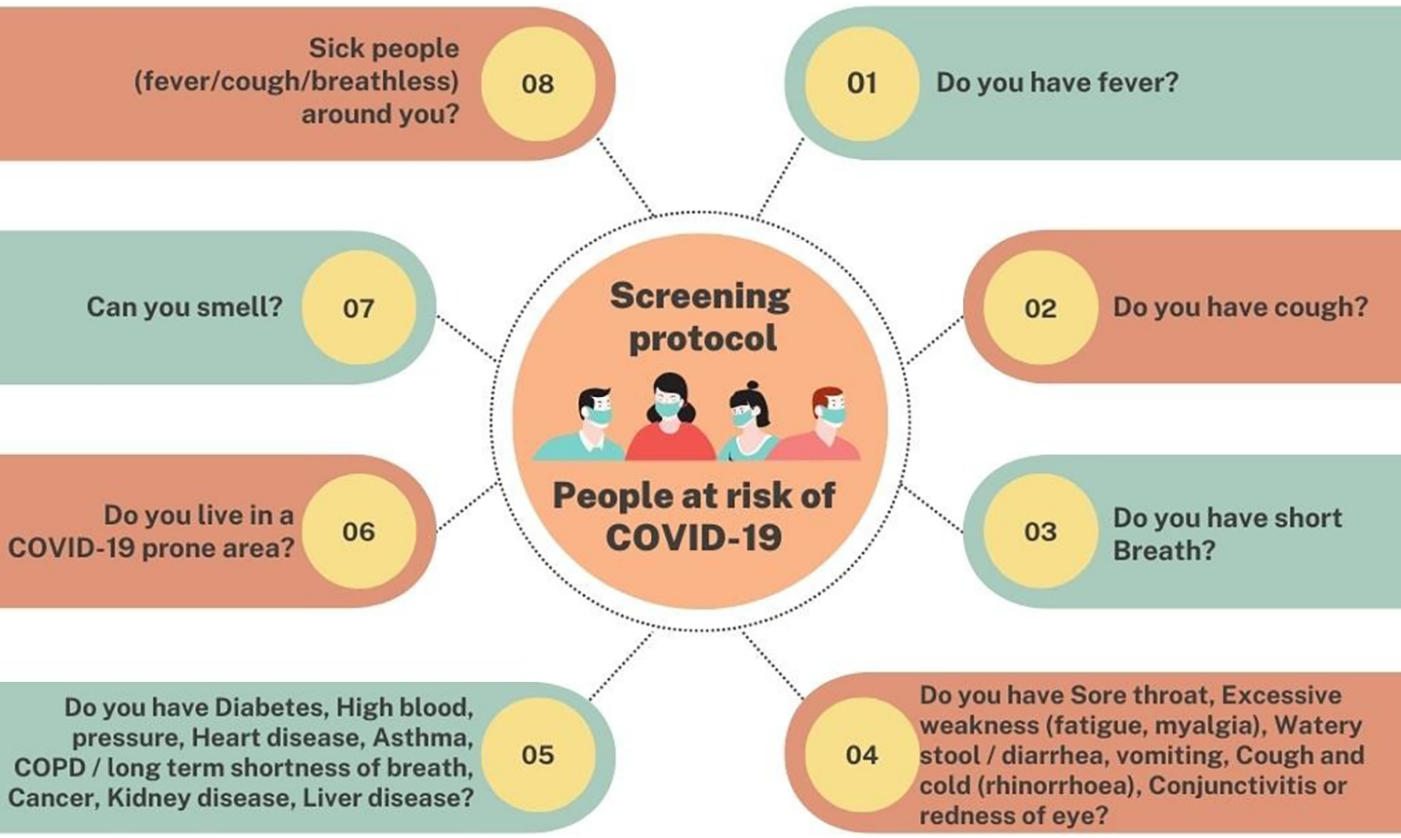
Risk assessment screening protocol.

The screening protocol was designed and developed by a group of healthcare experts consisting of the Directorate General of Health Services, Government of Bangladesh, Dhaka Medical College, Bangladesh, AIMS Lab, United International University, Bangladesh & CMED Health Limited, Bangladesh. The fundamental ideas were based on the COVID-19 WHO guideline and the following best practices from the BMJ. Before approaching any individual, healthcare professionals must verify their safety, wear masks, and wear personal protective equipment (PPE). The system also makes available information about the safety of healthcare professionals. The person being checked first has a digital health account made for them. The digital screening contained the questions in Figure 4.

**Figure 5:**
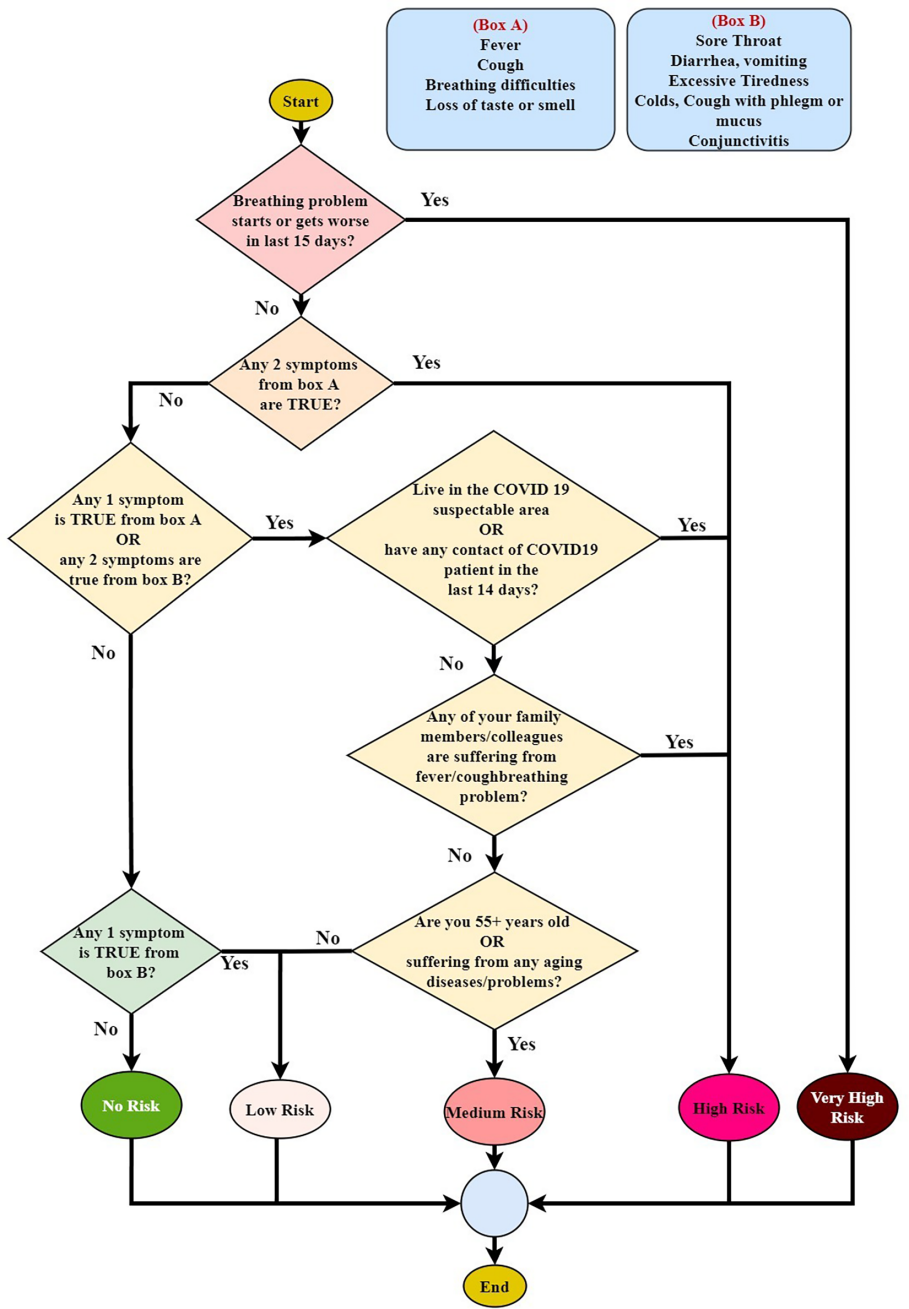
Risk assessment flow chart.

By examining the questionnaire, the screening results are demonstrated. Based on the outcome, there are five risk categories: no risk, low risk, medium risk, high risk, and very high risk. The methodology of analyzing the questionnaires is illustrated in Figure 5, where Box A and Box B stand in for the symptoms of each box. The matrix of the screening technique is shown in Table 1A. The procedure can be discovered to be based on five variables: Box A, Box B, Covid contact, Family, and 55+ years old/comorbid. The two symptom categories, Box A and Box B, may be identified from the questions in Figure 4. The method of determining the value of the Box A, Box B, and 55+ years old/comorbid status is shown in Tables 1B–D. The COVID-19 risk level is estimated in Table 1A using the calculated status of Box A, Box B, and 55+ years old/comorbid from Tables 1B–D. Table 1B calculates the Box A status by determining whether the person being tested has a fever, a cough, breathing difficulties, or loss of taste and smell, whether those issues have started or worsened over the past 15 days. All values are measured in Yes/No, with Yes represented by “1” and “No” represented by “0”. After obtaining the values for all of the symptoms, the values are summed up, and the associated status is set to 0, 1, 2, or 3. Table 1D determines whether the screened individual is over the age of 55, as well as whether he or she has any comorbidity like diabetes, high blood pressure, heart disease, asthma, Chronic Obstructive Pulmonary Disease (COPD) or long-term shortness of breath, cancer, renal disease, liver illness. Users are given advice based on their level of risk. People classified as “high risk” or “very high risk” are referred to the Upazila hospital for COVID-19 testing to confirm their status.

**Table 1A:** Risk assessment outcome matrix of the screening protocol.

Box A	Box B	Covid Contact	Family	55+ years old/Comorbid	Risk
0	0	Any	Any	Any	Safe
0	1	Any	Any	Any	Low Risk
0	2	0	0	0	Low Risk
0	2	0	0	1	Medium Risk
0	2	0	1	Any	High Risk
0	2	1	Any	Any	High Risk
1	Any	0	0	0	Low Risk
1	Any	0	0	1	Medium Risk
1	Any	0	1	Any	High Risk
1	Any	1	Any	Any	High Risk
2	Any	Any	Any	Any	High Risk
3	Any	Any	Any	Any	Very High Risk

**Table 1B:** Calculation the status of Box A symptoms.

Fever	Cough	Breathing difficulties	Breathing problem starts/increases since last 15 days	Loss of taste or smell	Sum	Status
0	0	0	0	0	0	0
0	0	0	0	1	1	1
0	0	1	0	0	1	1
0	0	1	1	0	2	3
0	0	1	0	1	2	2
0	0	1	1	1	3	3
0	1	0	0	0	1	1
0	1	0	0	1	2	2
0	1	1	0	0	2	2
0	1	1	1	0	3	3
0	1	1	0	1	3	2
0	1	1	1	1	4	3
1	0	0	0	0	1	1
1	0	0	0	1	2	2
1	0	1	0	0	2	2
1	0	1	1	0	3	3
1	0	1	0	1	3	2
1	0	1	1	1	4	3
1	1	0	0	0	2	2
1	1	0	0	1	3	2
1	1	1	0	0	3	2
1	1	1	1	0	4	3
1	1	1	0	1	4	2
1	1	1	1	1	5	3

**Table 1C:** Calculation the status of Box B symptoms.

Condition	Status
No Check	0
if any of Box B is positive	1
if any 2 of Box B is positive	2

**Table 1D:** Calculation the status of 55+ years old/comorbid.

>55	Comorbid	Status
0	0	0
0	1	1
1	0	1
1	1	1

### 2.2. System development and implementation

**Figure 6:**
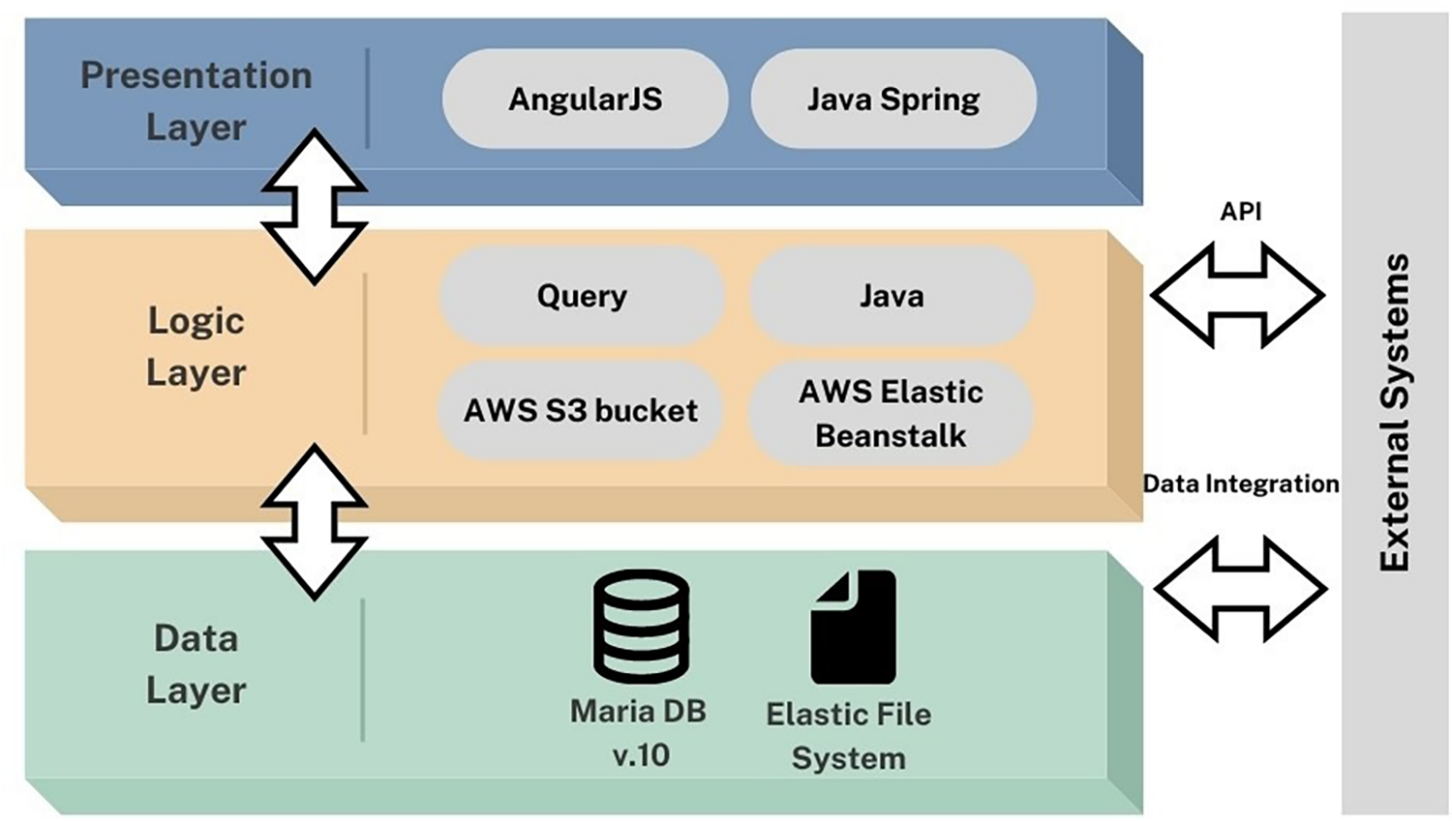
3-Tier architecture of the application.

The system was designed using a flexible architecture based on the organization's resources, such as software, platforms, and infrastructures, to allow for a swift response to changing situations. For example, introducing new symptoms and grading to the system in accordance with national and international guidelines. The system is built upon the Java Spring Framework to ensure data privacy, security, and reliability. Amazon Web Services (AWS) elastic beanstalk is used to load balance and auto-scale the backend. According to government rules, data can be shared with approved stakeholders using Application Programming Interfaces (API). AWS Linux and auto-scalable application servers such as load-balanced EC2 instances are used. Elastic File System (EFS) and S3 Buckets provide scalable data storage in the system. This enables the system to be highly customizable and configurable. The data analytics are based on data acquired by the system, which may dynamically generate customized charts and graphs. Black box testing approach was employed for the testing of the application. This method focuses on the functionality of the application and its features, without considering the internal structures and processes. The inputs were provided and the output was observed to determine if the application behaves as expected. The testing was performed in a passive manner, where the focus was on evaluating the results of the application's behavior. This approach helped to identify any issues related to compatibility, performance, and usability, and provided valuable insights into the overall functionality of the application. The three-tier architecture of the application is depicted in Figure 6.

#### 2.2.1. Database

The database used to store data collected from patient surveys was developed using the MariaDB (V.10) form of MySQL. The database deploys and maintains records of each person's information using an identifier that distinguishes each person's captured information by utilizing a unique identity. This is done by creating a digital health account for each assessed individual. The information is stored in real-time in the form of tables that can be retrieved based on the queries requested or required by the users *via* the dashboard.

#### 2.2.2. Information capturing and storage

The system can store and retrieve real-time screening information and outcomes, on-site or remotely, and both online and offline synchronization. Especially considering the environment of Bangladesh, where there is a lack of internet connectivity in many rural areas, the offline feature is necessary for smooth operation. At first, information from each person is stored in local or mobile storage. A cloud-based server, AWS allows data to be directly stored in the server *via* local storage in real-time to avoid information loss. At the same time, the system design is also efficient in storing the information in case of system disconnectivity, connection failure, or other network or internal issues. In such cases, the offline system would be functional. In the offline system, the application is designed to efficiently receive the upcoming information from each person and store it in local storage configuring as a part of application software. When the server connection is established, all stored information in the local storage will be updated in the AWS cloud database. The offline system scenario is significant in handling future system failures or disconnectivity issues. The data analysis will be performed in cases of an offline system in the local storage. Then, whenever the system gets an internet connection, it will automatically store it in the cloud.

#### 2.2.3. Mobile application and software

**Figure 7:**
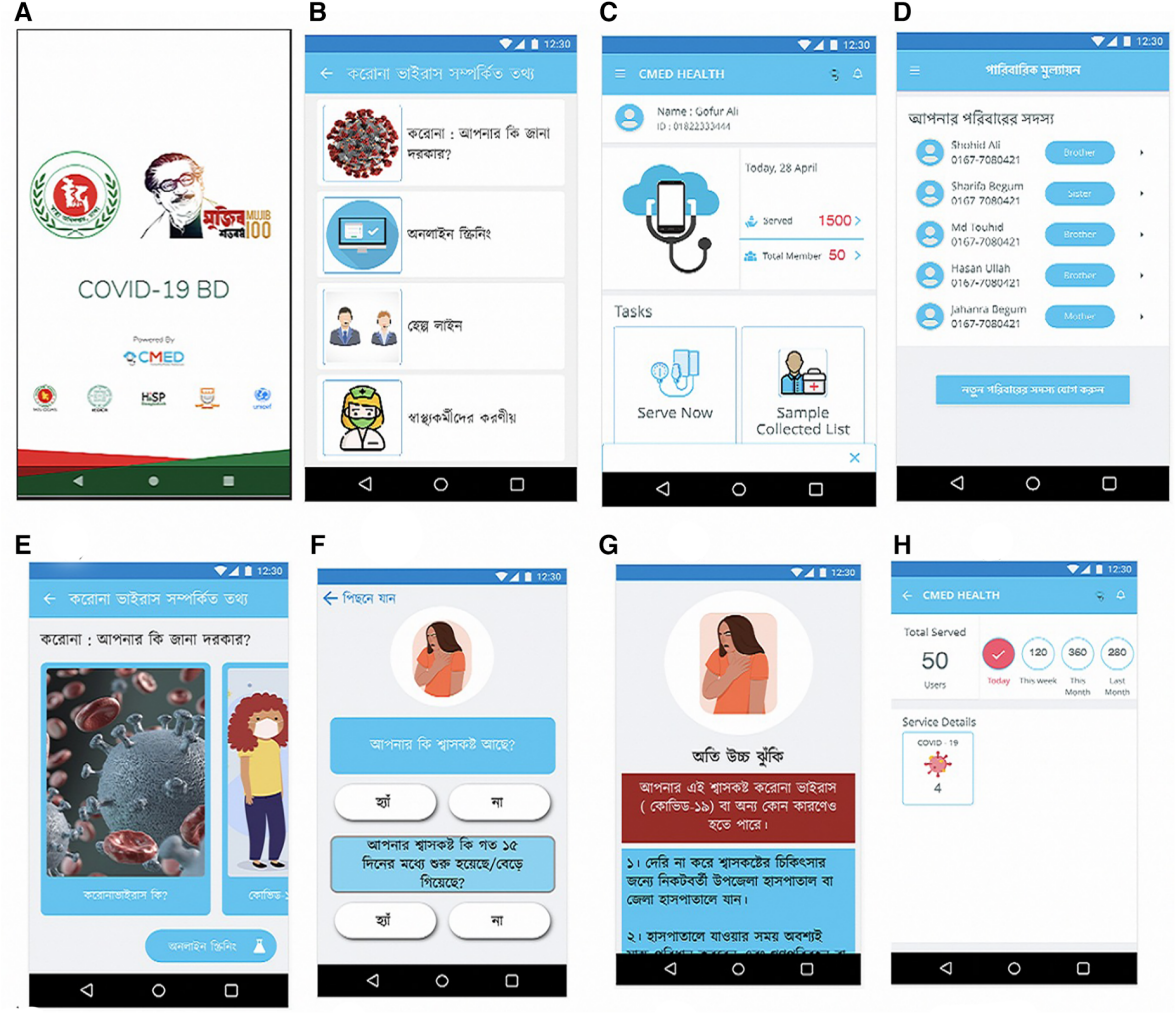
(**A**) Login page of the system; (**B**) services available to provide; (**C**) choose a specific person for screening for COVID-19; (**D**) list of the family members to select for screening next; (**E**) COVID-19 education; (**F**) sample question for COVID-19 screening; (**G**) screening outcome with essential advice what to do next; (**H**) health workers service providing statistics.

**Figure 8:**
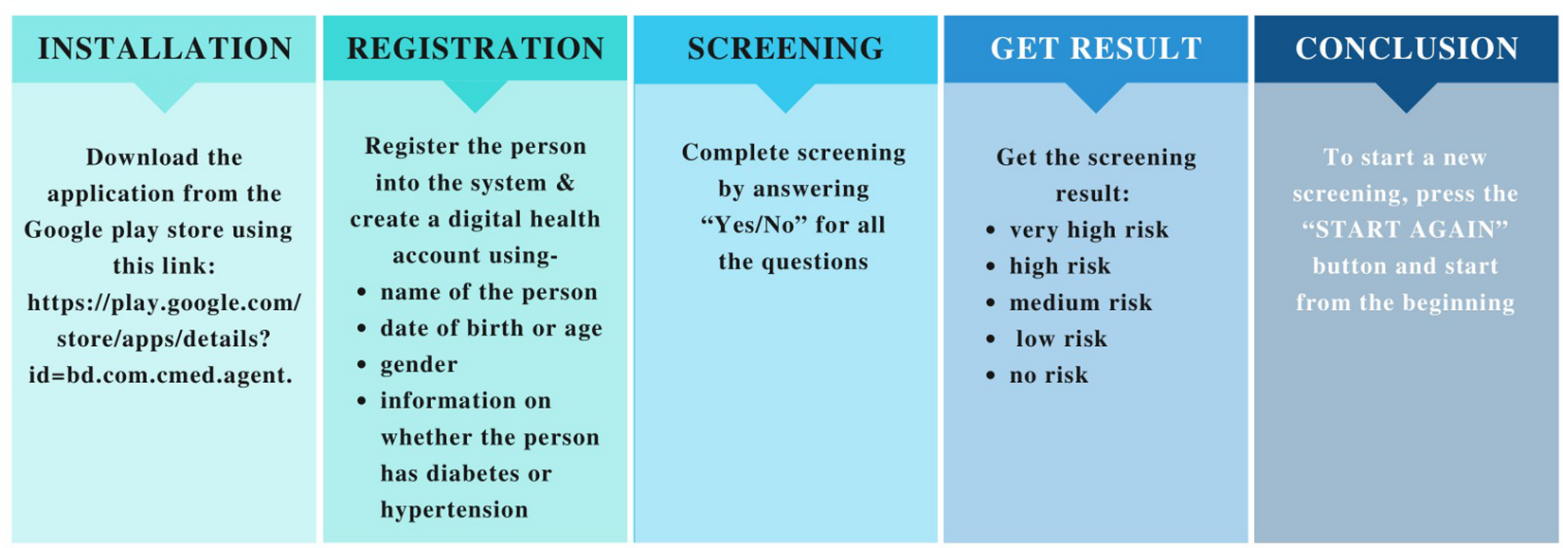
Steps of using the application.

A smartphone application (App) has been developed to screen and store real-time data. The application can be installed on a user's smartphone when a username and password are registered in the cloud. This application can generate COVID-19 screening-related information and results in 5 risk categories: no risk, low risk, medium risk, high risk, and very high risk. Healthcare personnel primarily use the software to screen community members and their families for COVID-19. The system will, however, uniquely identify each family member. Figure 7 depicts the App's features. The application enables role-restricted access to designated health workers. For example, a health worker assigned to screen in a specific region can only access information for people in that area as this is a national digital surveillance system, the DGHS of the government of Bangladesh has taken responsibility for acquiring users and keeping them involved. The steps for using the application are shown in Figure 8.

### 2.3. Functional features

#### 2.3.1. Educational module

Our application features a dedicated module for raising public awareness. Through our surveillance system, people can learn about the characteristics and transmission mechanisms of the COVID virus and how to independently manage their health and safety. The application supports both English and Bangla as a native language. Additionally, visual representations are offered for the better understanding of every person.

#### 2.3.2. Screening module

We created a reliable screening methodology and used consultative workshops to validate it. Screening is the main component of this monitoring system. Through a question and response process with a suspected user, the screening module performed risk prediction. It classifies the users according to five risk categories based on symptoms. Our surveillance system can perform COVID-19 screening in the community through registered health or community workers in their catchment areas.

#### 2.3.3. Monitoring and referral module

Through the government hotline number, our surveillance system connects a suspected patient for online consultation. Apart from that, it screens family members of potentially infected persons for contact tracing and connects a suspected case with government lab facilities for sample collection and testing. After screening, refer a positive case to a government-approved facility for further diagnostics, treatment, isolation, or hospitalization.

#### 2.3.4. Connected government module

Based on the acquired screening data, our surveillance system employs rule-based AI to forecast, classify, and visualize high-risk zones or epicenters of coronavirus, as well as to comprehend disease transmission dynamics for prevention. The system can do continuous data analysis to flatten the infection curve using various epidemiological models to assist the government in making lockdown decisions and resource planning. Furthermore, the data is shared with the government and policymakers in order to take action to limit the pandemic.

### 2.4. Data analysis and mapping

The collected data from the screening questionnaire was processed and analyzed using a rule-based AI system. The AI system used a set of predefined rules to categorize individuals based on their risk level of COVID-19 infection, as determined by their responses to the screening questionnaire. The rules-based AI system used a decision matrix, as shown in Table 1A, to categorize individuals into one of five risk levels: no risk, low risk, medium risk, high risk, and very high risk. After processing the data, the AI system mapped the results using a color-coded map that highlighted the distribution of risk levels in the screened population. This mapping allowed healthcare officials to visualize the spread of risk levels across different regions and take appropriate measures to contain the spread of COVID-19. In addition to mapping, the AI system generated personalized advice for each individual based on their risk level. Individuals categorized as high risk or very high risk were advised to seek testing at Upazila hospital to confirm their COVID-19 status. Overall, the rule-based AI system provided a fast and reliable method for analyzing and mapping the screening data, allowing healthcare officials to quickly identify high-risk individuals and take appropriate actions to prevent the spread of COVID-19.

## 3. Results: system outputs

**Figure 9:**
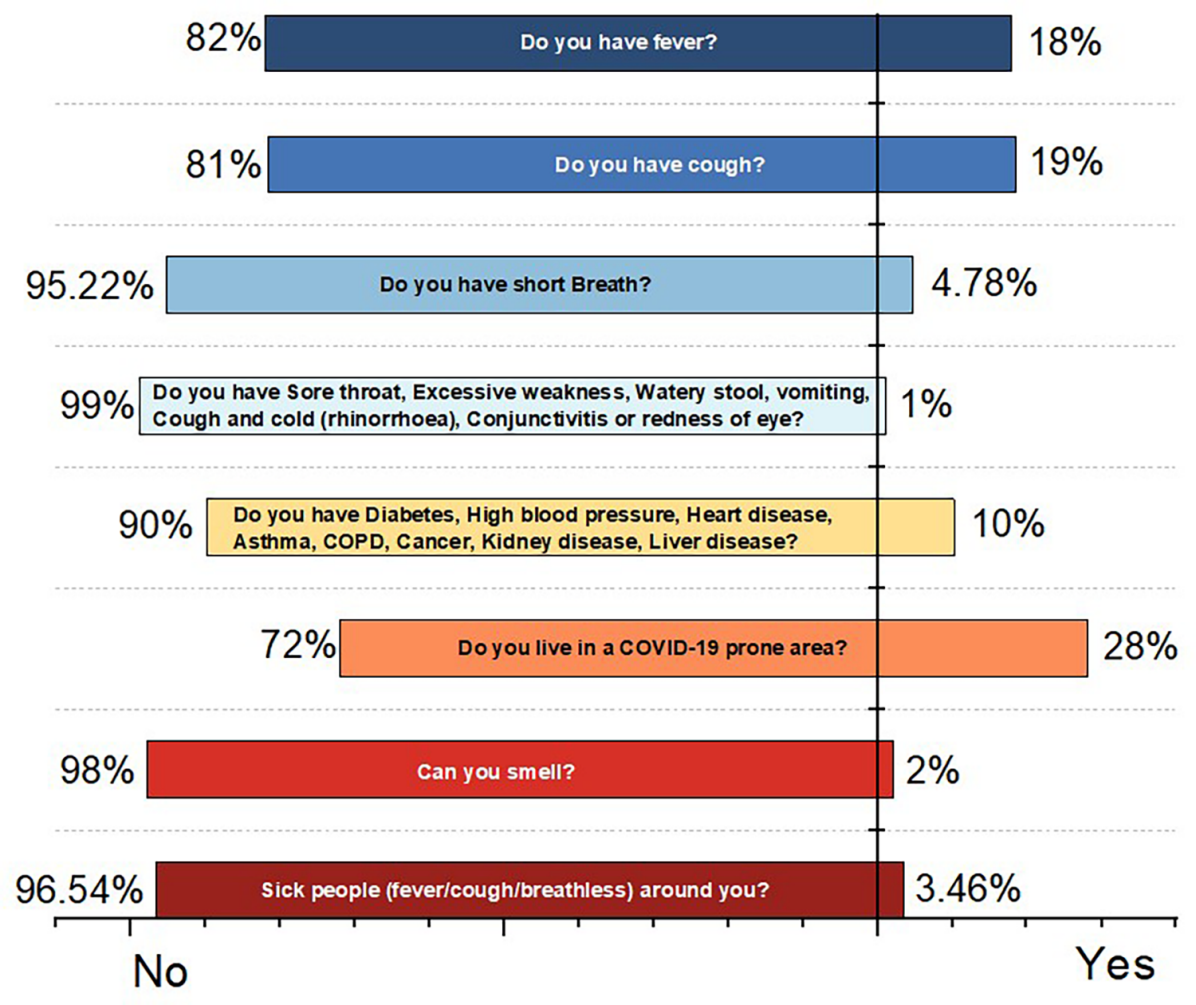
Measurement of COVID-19 questionnaire.

**Figure 10:**
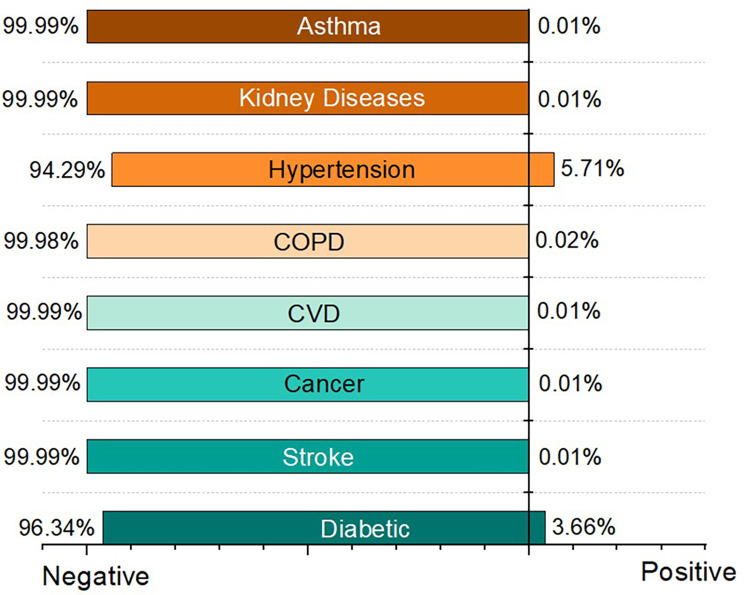
Measurement of comorbidities.

Our nationwide surveillance system was implemented in April 2020, and as of December 2022, it had screened a total of 1,980,323 cases. There were 957,162 males and 1,010,971 females screened, with the remainder being other genders. Users were required to respond to a questionnaire represented in Figure 4, with the answer options being yes or no. Figure 9 shows the result of this measurement. Other comorbidities are also taken into account by our system as a vital factor of COVID-19 risk. Figure 10 depicts the effects of comorbidities and indicates that hypertension is the most prevalent comorbidity among users, accounting for 5.71% of total screening. The second rank goes to diabetes, which accounts for approximately 3.66% of all users.

According to the data, approximately 51% of the overall screened populations are safe. Among the remaining 49% of the screened populations, 35% are low risk, 9% are high risk, 4% are mid risk, and the remaining 1% is very high risk. Furthermore, it displays the screened populations of these five categories based on their location (division/region). Table 2 displays the population in different divisions based on their tested outcome in five categories.

**Table 2:** The percentage of populations in different divisions according to their screened outcome in five different categories.

Very High Risk		High Risk		Mid Risk		Low Risk		No Risk	
Division/Region	Percentage of cases	Division/Region	Percentage of cases	Division/Region	Percentage of cases	Division/Region	Percentage of cases	Division/Region	Percentage of cases
Barishal	0.42	Barishal	0.23	Barishal	0.24	Barishal	0.27	Barishal	0.24
Chattogram	3.03	Chattogram	3.39	Chattogram	2.59	Chattogram	3.75	Chattogram	2.59
Dhaka	92.47	Dhaka	92.81	Dhaka	94.12	Dhaka	91.85	Dhaka	94.31
Khulna	1	Khulna	1.2	Khulna	0.73	Khulna	1.29	Khulna	0.71
Rajshahi	0.52	Rajshahi	0.43	Rajshahi	0.38	Rajshahi	0.5	Rajshahi	0.36
Rangpur	1.2	Rangpur	1.11	Rangpur	0.98	Rangpur	1.33	Rangpur	0.94
Sylhet	0.77	Sylhet	0.6	Sylhet	0.73	Sylhet	0.76	Sylhet	0.59
Mymensingh	0.57	Mymensingh	0.24	Mymensingh	0.24	Mymensingh	0.24	Mymensingh	0.25

**Figure 11:**
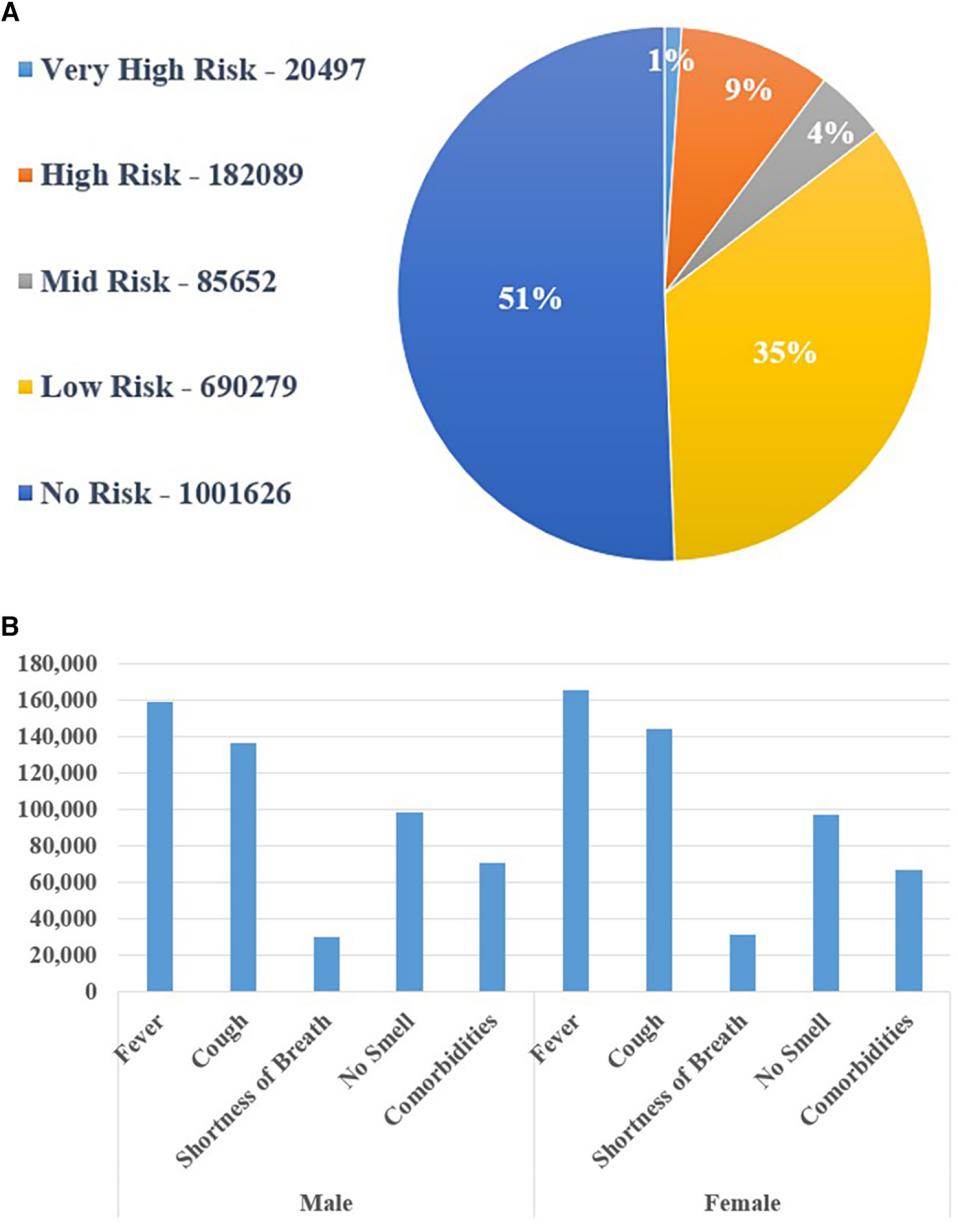
(**A**) screening outcome using the national COVID-19 digital surveillance system and (**B**) the proportion of people suffering from different symptoms.

According to the data, Dhaka has the most significant number of instances, whereas Mymensingh has the lowest number of cases in all five risk groups. It also displays the number of screened patients with various symptoms, such as fever, cough, shortness of breath, no-smell, contact with a positive case, and co-morbidities, as shown in Figure 11B.

The system can also determine the overall number of screened people with various symptoms based on age and gender. Figure 11A represents the percentage of people according to risk category and Figure 11B depicts the number of male and female patients with various symptoms. Females were shown to be more prone to fever and coughing than men. Furthermore, it has been discovered that the population aged 20 to 49 years was more afflicted by various COVID-19 symptoms. The results can be viewed at multiple levels of the Bangladeshi administrative system, including the district, Upazila (a sub-unit of a district), union (the smallest rural administrative and local government unit), and ward (optional division of a city or town for administrative and representative purposes). To make the system more user-friendly, several report options from a dropdown list can be selected based on the user's demands.

## 4. Discussion

In this study, we developed a nationwide web-based digital surveillance system that collects COVID-19 data nationwide on one platform and identifies COVID-19 risky cases. The system employs rule-based AI and can provide insights to relevant government and private entities in order to take the required steps to avoid the spread of COVID-19 in Bangladesh. The Center for Systems Science and Engineering at Johns Hopkins University created a web-based dashboard to visualize and track COVID-19 instances in real-time worldwide (37). Similarly, the WHO revealed its COVID-19 dashboard, which also maps and lists COVID-19 cases and the total number of deaths by country and Chinese province, with information panels about the map and its data resources (38). While this dashboard provides and updates COVID-19 cases for Bangladesh, it does not provide cases by city or data about screening, outcomes, and follow-up data, which are essential to track progress. A recent study in Brazil suggested that participatory surveillance can complement traditional approaches by adding real-time spatial data to detect priority participants for COVID-19 testing (13). Another study in China showed the usefulness of an online questionnaire for COVID-19 surveillance, which provided data on trends and supported identifying risk factors (18). Sweden developed SmiNet-2, an internet-based surveillance system for communicable diseases that automatically merges clinical and laboratory notifications to case records (39). SmiNet-2 used tools for outbreak investigations, contact tracing, and case management, which are also included in our surveillance system. These studies suggest that our digital surveillance system might be a valuable adjunct to traditional approaches for COVID-19 surveillance in Bangladesh.

Previous studies have shown that involving community-level health workers and linking with local health centers have improved several health outcomes (40–42). A study in Nepal suggested that community health worker-collected mobile phone data as a possible adjunct to surveillance for diarrhea in a resource-limited setting (14). Taiwan developed a mobile medical station for the immediate treatment and screening of covid affected individuals (43). We collaborated with the DGHS in Bangladesh to implement a rule-based AI COVID-19 digital surveillance platform for community mobilization in urban and rural areas, involving over 13,700 community clinics, 138 urban primary healthcare centers, and over 30,000 government and non-government health workers or social volunteers. To date, it is the most powerful technical solution for screening, tracking, and collecting COVID-19 samples for testing in Bangladesh. In addition, our system enables healthcare practitioners to refer and educate people about COVID-19. A central monitoring system enables adequate resource planning and allocation. In Iran, a COVID-19 surveillance system employed geographical information systems to create epidemiological maps of cases and incidence rates by province; however, that system does not link data with healthcare providers and centers (44).

Several digital health tools have recently been developed in response to COVID-19 worldwide. In a study, a chatbot was developed for the rapid detection of COVID-19 using symptoms (45). A deep learning-based algorithm was developed to detect the COVID from respiratory sound recording utilizing a mobile device (46). Based on the data, it determines the severity and performs screening. An IOT-based “Smart COVID-Shield” was designed to detect common symptoms such as fever and coughing while also ensuring social distance standards are met (47). Moreover, an electronic health record-based surveillance system has been developed in the United States, which supports scripted triaging, electronic check-in, standard ordering and documentation, secure messaging, real-time data analytics, and telemedicine capabilities (48). China's National Health Commission developed a “close contact detector” application/platform that uses big data from government authorities about the movement of people and disease case records. Based on their location and recent movements, the platform can inform the user whether they have been in contact with a person confirmed or suspected to have the virus within the last two weeks and can be accessed by popular social media platforms (38). Compared to this, our system currently cannot follow up on a patient till the outcome (death/cured).

Moreover, the surveillance system can be adapted for other health conditions or countries requiring digital surveillance. Previous studies in Bangladesh have shown the use of mobile phone services for health in Bangladesh and the willingness of people to use the systems (49–51). Our digital COVID-19 surveillance system is intuitive, simple, and easy to understand, which could be integrated with COVID-19 telemedicine services (52) and scaled up for use by the general population. The system was designed for the local context, with both textual and video tutorials. Short training was delivered to healthcare support providers in their native language, ensuring simplicity of use and faster service delivery.

Digital health system captures and stores personal health information; therefore, data privacy and security are a big concern. This is true for our system, where many users will access a shared system to capture and store information. In our system, we dealt with privacy concerns by providing user-level access restrictions and control, regulatory considerations such as having completed privacy impact assessments on the platform, and process considerations such as centralized control over the ability to add new users and control permissions, as well as privacy training. The AWS Cloud Platform provides several layers of encryption to protect user data. Multiple third-party security assessments have been completed, and the development team is experienced in deploying big health projects.

There are some limitations to this system. First, the system is not integrated into existing infrastructure, such as hospital systems, through any application program interface (API). This was done to avoid delays related to developing the APIs and other compatibility issues but could be implemented when it becomes advisable. Due to the lack of such integrations, specific actions like patient hospitalization require manual data reconciliation instead of the ideal automated state triggered through software. Second, although the government of Bangladesh has launched the system nationwide to screen all populations, in many areas of Bangladesh, especially in rural areas, there is a lack of skilled healthcare workers to use technology. However, we provided training to all healthcare workers in the community clinic in Bangladesh.

This is the country's first practical and trustworthy digital surveillance system for COVID-19, and it has received overwhelming acceptance. Our well-developed system performed admirably for COVID-19 screening based only on symptoms. Our protocol succeeded because it was based on WHO and BMJ guidelines and an expert consultative session. Besides a robust infrastructure, such as well-developed mobile and web applications, the surveillance system is user-friendly and reliable for both users and the government. Moreover, this system offers a wide range of user input options, such as self-use, use through a health worker, or even *via* a phone call. These make the system available to all types of users and every region of the country where connectivity is inadequate. This system also includes an awareness module where users can learn about COVID-19 and its transmission mechanism. This aided in reducing the number of affected cases and the severity of COVID-19 among people. Based on a thorough literature review, we attempted to develop a system that would maintain the worldwide standard. We did not encounter any major infrastructure challenges while developing our system. Our experienced team thoroughly tested the infrastructure to ensure its proper implementation, resulting in no issues with accessing the system from an infrastructure perspective. We utilized AWS Elastic Beanstalk for handling concurrent requests and load balancing, and MariaDB Version 10 (a form of MySQL) for our database. Our goal was to ensure that our system was robust, reliable, and scalable to meet the needs of our users. As the primary objective during the development of the application was to effectively screen and estimate the risk of COVID-19, we were unable to conduct an impact evaluation of the application's ease of use due to government policy restrictions. However, the system was designed with the capability to track actual clinical risk outcomes, allowing us to monitor the accuracy of our risk stratification. This could be the possible area of future study. Although, government policies could be one of the challenges to implement in such a system. This is the only well-established digital COVID-19 surveillance system in Bangladesh that connects people, government, and policymakers.

## 5. Conclusion

Our digital surveillance system enables rapid COVID-19 screening and presents results based on risk categories with the help of rule-based AI. For a confirmed COVID-19 diagnosis, the screened High Risk and Very High-Risk cases are forwarded to the next level of care. Our systems can complement the traditional surveillance system in Bangladesh to reach broad population groups in a crisis. This monitoring system can benefit the wider populace by providing a quick and cost-effective approach to screening COVID-19. Further system development to store test results, track positive cases, and use AI to generate insights might help local and national planning. This system can be instrumental in helping the government to develop a future national digital health surveillance system for other conditions.

## Data Availability

The raw data supporting the conclusions of this article will be made available by the authors, upon request.
